# The Influence of Music on Prefrontal Cortex during Episodic Encoding and Retrieval of Verbal Information: A Multichannel fNIRS Study

**DOI:** 10.1155/2015/707625

**Published:** 2015-10-05

**Authors:** Laura Ferreri, Emmanuel Bigand, Patrick Bard, Aurélia Bugaiska

**Affiliations:** UMR-CNRS 5022 “Laboratoire d'Etude de l'Apprentissage et du Développement”, Université de Bourgogne, 21000 Dijon, France

## Abstract

Music can be thought of as a complex stimulus able to enrich the encoding of an event thus boosting its subsequent retrieval. However, several findings suggest that music can also interfere with memory performance. A better understanding of the behavioral and neural processes involved can substantially improve knowledge and shed new light on the most efficient music-based interventions. Based on fNIRS studies on music, episodic encoding, and the dorsolateral prefrontal cortex (PFC), this work aims to extend previous findings by monitoring the entire lateral PFC during both encoding and retrieval of verbal material. Nineteen participants were asked to encode lists of words presented with either background music or silence and subsequently tested during a free recall task. Meanwhile, their PFC was monitored using a 48-channel fNIRS system. Behavioral results showed greater chunking of words under the music condition, suggesting the employment of associative strategies for items encoded with music. fNIRS results showed that music provided a less demanding way of modulating both episodic encoding and retrieval, with a general prefrontal decreased activity under the music versus silence condition. This suggests that music-related memory processes rely on specific neural mechanisms and that music can positively influence both episodic encoding and retrieval of verbal information.

## 1. Introduction

Episodic memory can be defined as a neurocognitive system, uniquely different from other memory systems, which enables human beings to remember past experiences [1]. Numerous studies have investigated the factors that can boost this system. According to the encoding specificity principle [2], the memory trace of an event and hence the properties of effective retrieval cues are determined by the specific encoding operations performed by the system on the input stimuli. Craik and Lockhart [3] first proposed that the durability of the trace depends on the “depth” of encoding processing, deeper semantic processing allowing better encoding of the target information. Furthermore, it has been demonstrated that the encoding context of an event plays a crucial role in successful memory performance. For instance, a rich context given by stimuli with a high (positive or negative) emotional valence can enhance the encoding of contextual information associated with an item [4]. In this scenario, music could offer a perfect example of an enriched context. Indeed, given its complexity as a stimulus that evolves through time and has a strong emotional impact [5], music is likely to enrich the encoding context of an event, thereby improving subsequent memory performance. The evocative power of music is fascinating and undisputed: it can evoke both emotional states and personal events from the past [6]. Several studies have revealed a specific episodic memory for music, showing how it depends largely on emotion [7] and revealing the existence of specific related neural processes [8]. Nevertheless, the question of whether music as an encoding context can enhance episodic memory performance, especially concerning verbal material, remains debatable and controversial. Several studies have shown that music, presented either as background or as sung text, can enhance verbal learning and memory in both healthy and clinical populations [9–13]. However, several authors have recently claimed that music can also draw attention away from the to-be-remembered information, thus interfering in memory performance [14–16]. The key to solving this question seems to rely on a better understanding of the processes involved: improving our knowledge of how music can boost memory performance at both behavioral and functional (i.e., neuronal) levels could shed new and essential light on the most efficient music-based paradigms and interventions.

In a series of functional near-infrared spectroscopy (fNIRS) studies, we previously showed that background music during the episodic encoding of verbal material can improve item and source memory performance and modulate prefrontal cortex (PFC) activity [10, 11]. More specifically, fNIRS studies have found that music leads to decreased activation (i.e., decrease in oxyhemoglobin-O_2_Hb and deoxyhemoglobin–HHb increase) in the dorsolateral prefrontal cortex (DLPFC), known to be important for organizational, associative, and memory encoding [17]. In view of fNIRS studies showing decreased PFC activity during verbal learning in which subjects were helped during their performance [18, 19], we hypothesized that music could modulate episodic encoding by modifying the need of extra organizational and strategic encoding usually attributed to the DLPFC [20] and facilitating the creation of richer associative bindings crucial for subsequent retrieval [10, 11]. However, both methodological and theoretical caveats raise important issues. The present work therefore aims to increase our knowledge of music-related memory processes by extending investigations of background music and verbal memory through three main questions arising from these previous studies.

First, existing fNIRS data are limited to the encoding phase, raising the question of which mechanisms are involved during episodic retrieval. Research on episodic memory has clearly demonstrated that in order to understand how memories are formed, we need first to understand many cognitive and neurobiological processes involved in both encoding and retrieval, as well as the interactions among these phases [21]. Furthermore, in the light of the contrasting results in the literature, it is crucial to know whether the music facilitation reflected in decreased PFC activation is also found in the retrieval phase or whether by contrast it shows a more demanding pattern in line with the interference hypothesis. Therefore, in the present study, fNIRS prefrontal data were acquired during both encoding and retrieval of words in order to test the hypothesis that the PFC disengagement found during memory formation is also found during the retrieval phase.

Secondly, previous fNIRS acquisitions were limited to eight channels covering the bilateral DLPFC, thus hindering the possibility of ascertaining the music effect throughout the lateral prefrontal cortex, which is crucial during episodic memory processes [22–24]. Ventrolateral and dorsolateral regions of the PFC have been shown to implement different controls that provide complementary support for long-term memory encoding and retrieval. More specifically, during the encoding phase, ventrolateral prefrontal cortex (VLPFC) regions contribute to the ability to select goal-relevant item information and strengthen the representation of goal-relevant features, while DLPFC regions contribute to memory enhancement by allowing associations among items in long-term memory during encoding [17]. Concerning the retrieval phase, several studies on episodic memory retrieval have found a fronto-parieto-cerebellar network, in which several bilateral frontal regions seem to mediate processes that act in the output of episodic retrieval operations (see [22] for a review). It is therefore important to understand whether the observed PFC deactivation is restricted to the dorsolateral region or whether it includes the whole lateral prefrontal cortex. While a delimited prefrontal deactivation would suggest that music specifically modulates certain cognitive processes, a decrease throughout the PFC during the music condition would indicate an overall PFC disengagement and suggest that music-related memory processes rely on music-specific and unusual neural mechanisms. Hence, in the present study, a multichannel (i.e., 48 measurement points) fNIRS system was used to monitor the whole PFC cortex during episodic encoding and retrieval.

The third important point concerns a behavioral issue. Our previous behavioral and functional results led us to explain the findings in terms of associative bindings. A musical context may afford efficient mnemonic strategies allowing the creation of interitem and item-source associations that can help subsequent retrieval. These mnemonic strategies would result in PFC deactivation [18, 19]. If confirmed, this would be an important contribution to the existing debate about the complex music-memory issue. However, previous studies used judgment memory tasks, whereby subjects were presented with a copy of old items and had to retrieve and judge whether or not each item had been presented previously (item memory) and, if so, in which context (source memory). Using this paradigm, it was not possible to investigate possible associative processes. Therefore, in the present study we used a free recall task in order to investigate if subjects adopted particular strategies during the retrieval phase.

To extend our knowledge of music-related memory processes and contribute to the current debate, the present study used multichannel fNIRS to test lateral prefrontal activations during music-related encoding and retrieval (i.e., free recall). We asked subjects to memorize lists of words, presented with a background of either music or silence, and to retrieve as many words as possible after an interference phase. We used a 48-channel fNIRS system to monitor their PFC activity bilaterally. Based on the hypothesis that a background of music would modulate PFC activity throughout the memory processing stages, we expected to find less cortical activation during both the encoding and the retrieval phases under the music condition, in line with our previous studies on verbal encoding with music [10, 11]. Furthermore, clustered retrieval of previously encoded words for the music condition when compared to the silence condition would suggest that music helps encoding through the implementation of associative strategies.

## 2. Materials and Methods

### 2.1. Participants

Nineteen young healthy students at the University of Burgundy (13 female, mean age 21.65 ± 3.2 years) took part in the experiment in exchange for course credits. All the participants were right-handed, nonmusicians, and native French-speakers and reported having normal or corrected-to-normal vision and hearing. None were taking medication known to affect the central nervous system. Informed written consent was obtained from all participants prior to taking part in the experiment. The study was anonymous and fully complied with the Helsinki Declaration, Convention of the Council of Europe on Human Rights and Biomedicine.

### 2.2. Experimental Procedure

Subjects were seated in front of a computer in a quiet, dimly lit room. After the 48 fNIRS probe-set had been fitted on the forehead overlying the PFC (see fNIRS section below for detailed description), the in-ear headphones inserted and the sound recorder placed, subjects were informed that they would be presented with different lists of words with two auditory backgrounds: music or silence. They were asked to memorize the lists of words and were told that, after a brief backward counting task, they should mentally recall the previously seen words and then say as many as they could.

Verbal stimuli consisted of 90 taxonomically unrelated concrete nouns selected from the French “Lexique” database ([25]; http://www.lexique.org/). Words were randomly divided into six lists (15 words per list, 45 words for each condition), matched for word length and occurrence frequency. In the music condition, the background music used in all blocks was the instrumental jazz piece “Crab walk” (by Everything But The Girl, 1994). This excerpt was chosen after a pretest among a list of 8 pieces representing different musical genres (such as classical, jazz, new age) preselected by the authors. All the excerpts were instrumental in order to avoid possible interference between the lyrics and the verbal material to be encoded. The excerpts were evaluated by nonmusician participants in terms of arousal, emotional valence, and pleasantness using a 10-point scale. Participants were also asked to report if the music was familiar or not. The selected piece was chosen for its positive valence, medium arousal quality and for being rated as pleasant and unfamiliar.

Three blocks for each condition (music or silence) were presented to each subject, giving a total of 6 experimental blocks. Each block consisted of three phases, namely, encoding, interference, and retrieval. In the encoding phase, 15 words were displayed successively against a background of music or silence. The auditory stimulation started 15 s before the first word was displayed, continued during the sequential display of words, and ended 15 s after the last word. Words in each block were displayed for 3 s per word, amounting to 45 s for the sequential presentation of 15 words. Each encoding phase therefore lasted 75 s (15 s background, 45 s words, and 15 s background). Verbal stimuli were visually presented in white on black background in the middle of the screen. Auditory stimuli were presented using in-ear headphones, and the overall loudness of the excerpts was adjusted subjectively to ensure a constant loudness throughout the experiment.

Prior to the retrieval phase, subjects were asked to count down from a given number displayed on the screen until the word “stop” appeared. The interference phase lasted 30 seconds.

**Figure 1:**
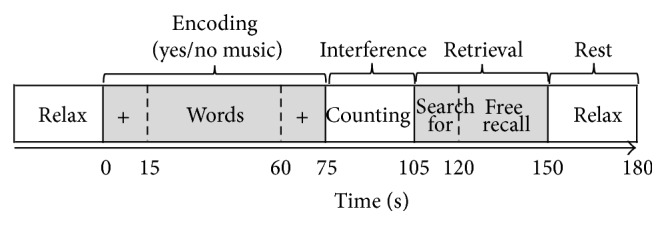
Representation of one encoding-interference-retrieval block between two 30 s rest blocks. Each block consisted of 15 s of context (+ on the screen) alone (music or silence in the earphones), then 45 s of context and word encoding (with either background music or silence), and then again 15 s of context (+) alone. After 30 seconds of interference phase (counting), subjects were asked to search for previously encoded words (search for, with either background music or silence, 15 s) and then to recall as many words as they could (free recall, 30 s).

The retrieval phase was divided into 15 s of a “search for” phase, in which the previous encoding background was presented in the earphones and subjects were asked to mentally recall the previously seen words and 30 s of a “free recall” phase, in which subjects were asked to say aloud as many words of the previously encoded list as possible. There were two reasons for this procedure: first, to resituate the subjects in the source of encoding, enabling good memory performance (see, e.g., [26, 27]); secondly, to have a control condition for possible voice-movement artifacts. A sound recorder was used to record subjects' free recall performance. The retrieval phase lasted 45 s. The total duration of each block was 3 minutes. Each block was followed by a 30 s rest (silent) (Figure 1).

The order of music/silence blocks was counterbalanced, as well as the order of word lists and the order of words in the lists. During the rest periods, subjects were instructed to try to relax and not to think about the task; in contrast, during the context-only phases of the blocks, participants were instructed to concentrate on a fixation cross on the screen and to focus on the task. Presentations of task instructions and stimuli were controlled by E-Prime software (Psychology Software Tools, Inc.) using a laptop with a 15′′ monitor. The entire experimental session, including fNIRS recording, lasted about 20 minutes.

### 2.3. fNIRS Measurements

**Figure 2:**
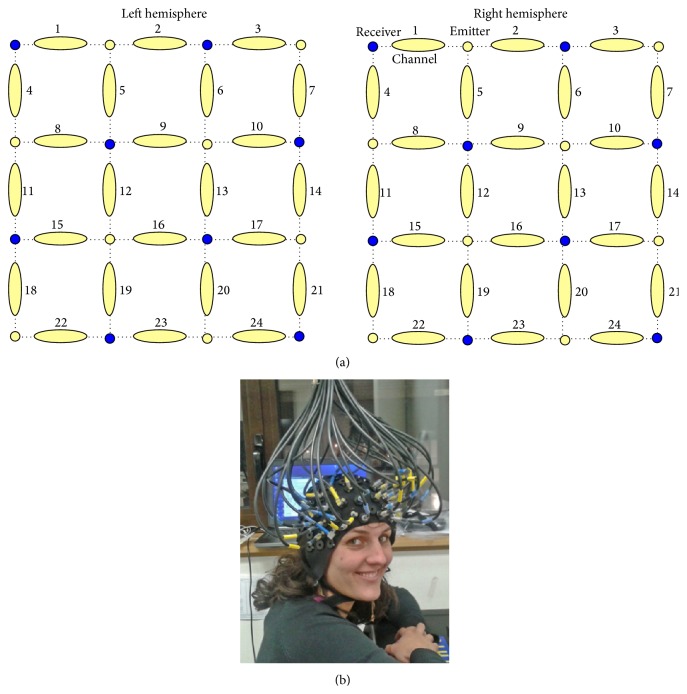
Channels template (a) and localization (b). The 48-channel NIRS system consisted in 8 emitters (yellow circles) and 8 receivers (blue circles) for each hemisphere, resulting in 24-left and 24-right measurement points (yellow lengthened shapes).

A 48-channel fNIRS system (OxymonMkIII, Artinis Medical Systems B.V., The Netherlands) was used to measure the concentration changes of O_2_Hb and HHb (expressed in *μ*M) using an age-dependent constant differential path-length factor given by 4.99 + 0.0067*∗*(age^0.814^) [28]. Data were acquired at a sampling frequency of 10 Hz. The 48 fNIRS optodes (24 emitters and 24 detectors, Figure 2(a)) were placed symmetrically over the lateral PFC. The distance between each emitter and detector was fixed at 3 cm. For each hemisphere, fNIRS channels measured the hemoglobin concentration changes at 24 measurement points in a 12 cm^2^ area, with the lowest optodes positioned along the Fp1-Fp2 line and the most central optodes 2 cm from the Cz line [29], in accordance with the international 10/20 system [30]. From top to bottom, these measurement points were labeled 1–24 (see Figure 2(a)).

To optimize signal-to-noise ratio during the fNIRS recording, the 48 optodes were masked from ambient light by a black plastic cap that was kept in contact with the scalp with elastic straps, and all cables were suspended from the ceiling to minimize movement artifacts [31] (Figure 2(b)). During data collection, O_2_Hb and HHb concentration changes were displayed in real time, and the signal quality and the absence of movement artifacts were verified.

### 2.4. Data Analysis

#### 2.4.1. Behavioral Data

Memory performance was calculated for each subject under both conditions by computing the total number of correctly retrieved words. Incorrectly retrieved items were considered as intrusions. Paired *t*-tests were used to compare the free recall memory and intrusion scores in the silence and music conditions. Subjects' possible associative strategies at encoding were examined using cluster analysis, in which the chunks created at retrieval indicated the level of interitem associations at the encoding phase. We therefore calculated the number of items presented in a row (i.e., one following the other) during the encoding phase that were retrieved in chunks, identifying 2-, 3-, 4-, 5-, and 6-word chunks produced by each subject and under each condition (e.g., if the subject encoded “bottle,” “fork,” “match,” “coat,” and “pool” in the encoding phase and then subsequently retrieved “fork,” “match,” and “coat” during the free recall task, this constituted a 3-word chunk; if the subject retrieved “bottle,” “match,” and “coat,” this constituted a 2-word chunk). Paired *t*-tests successively compared the most consistent chunks (>2-words) over the total of chunk ratios between the silence and music conditions.

#### 2.4.2. fNIRS Data

In order to eliminate task-irrelevant systemic physiological oscillations, the O_2_Hb and HHb signals were first low-pass filtered (5th-order digital Butterworth filter with cut-off frequency 0.1 Hz) for each of the 48 fNIRS measurement points.

To determine the amount of activation during the encoding phase for the two conditions, data in each of the 6 experimental blocks was baseline corrected using the mean of the O_2_Hb and HHb signals during the last 5 s of the rest phase. We then sample-to-sample averaged (i.e., 10 samples/s) the baseline-corrected signals over the 3 blocks of each condition, yielding one average music and silence O_2_Hb and HHb signal per participant for both the encoding phase and the retrieval phase (both “search for” and “free recall” tasks). We then computed the maximum O_2_Hb and the minimum HHb delta-from-baseline values over the 45 s (for the encoding), 15 s (for the “search for” retrieval), and 30 s (for the “free recall” retrieval) stimulus windows, for both the music and silence average block of each participant and for each channel (see Figure 4). Delta values were then statistically analyzed using a repeated measure ANOVA with 2 (music/silence condition) × 2 (left/right hemisphere) × 24 (optodes) repeated factors. Paired *t*-tests were also used to compare each channel as well as the means of left right activity for the silence and music condition and for each phase of the memory task [31] (see Figure 4).

Furthermore, in order to ascertain the PFC activation during the entire block of music/silence encoding and retrieval conditions, we ran a complete group time-series analysis in which we averaged O_2_Hb, HHb, and total Hb (THb) concentrations over 5 s windows (i.e., one average point for each 5 s) all over the blocks of encoding, interference, “search for,” and free recall phase, getting 35 successive measures of concentrations. Time-series data were then analyzed using a repeated measure ANOVA with 2 (music/silence condition) × 2 (left/right hemisphere) × 24 (optodes) × 35 (points in time) within-subject factors.

## 3. Results

### 3.1. Behavioral Results

**Figure 3:**
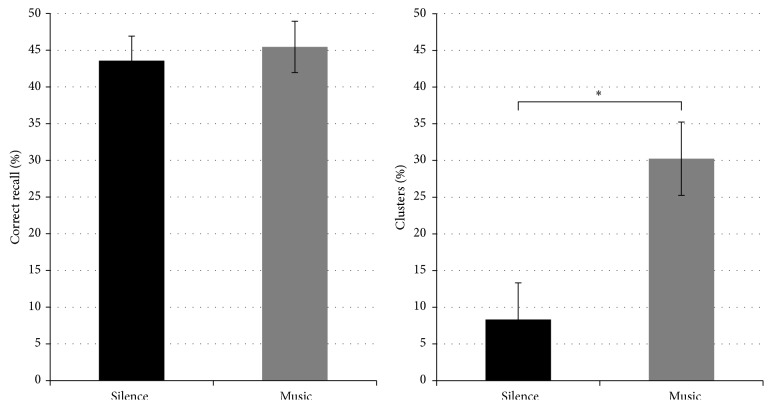
Behavioral results showing the total percentage of correctly retrieved words (left side) and of clusters (>2-word chunks) created in the free recall phase for the silence (black bars) and music (grey bars) conditions. *∗* shows statistically significant differences (*P* < .05).

Paired *t*-tests on the free recall memory performance and intrusion scores revealed no differences in the total number of correctly retrieved words and false-alarm rates between the music and silence conditions (*t*(18) = 1.17, *P* > .05). However, cluster analysis revealed a significant difference between the two conditions concerning the number of chunks created at retrieval. While *t*-tests on the total number of words retrieved in chunks did not reveal a significant difference between the two conditions (*t*(18) = −.165, *P* > .05), a significant difference was found for cluster creation, data revealing that subjects created more consistent chunks (>2 words) in the music than in the silence condition (*t*(18) = 2.23, *P* = .02) (Figure 3).

### 3.2. fNIRS Results

**Figure 4:**
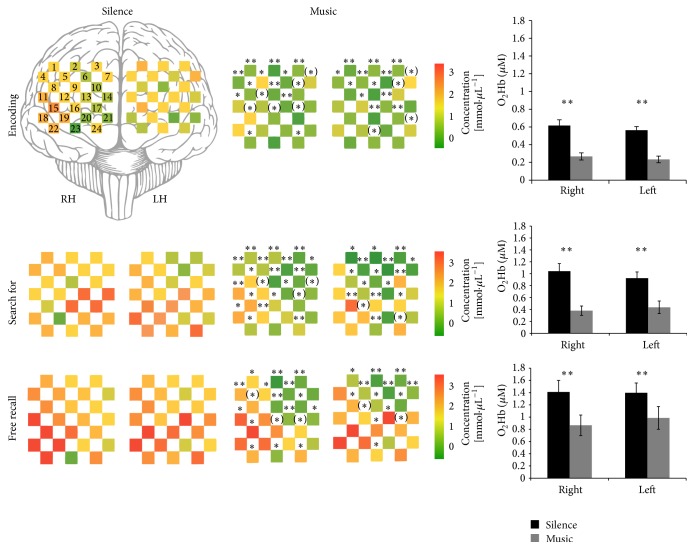
O_2_Hb Δ-to baseline values expressed in micromoles for silence (left side) and music (right side) conditions, for all 48 channels (24 right -RH-, 24 left -LH-), during the encoding, “search for” and free recall phases. Red = more activated; green = less activated. The whole prefrontal cortex resulted significantly less activated in the music condition during the three phases. *∗∗*, *∗*, and (*∗*) on the music channels show statistically significant differences (resp., *P* < .01, *P* < .05, and .05 < *P* < .09) between the two conditions for each channel. The difference between the two conditions is also showed in the left right of the figure, with black and grey bars representing O_2_Hb Δ-to baseline mean values, respectively, for silence and music conditions, in the right and left hemisphere (*∗∗* for *P* < .01 resulted from paired *t*-tests comparisons).

Figure 4 shows a channel-level analysis on O_2_Hb delta-to-baseline values for each phase of the memory task (encoding, “search for,” and free recall). The repeated-measures ANOVA on O_2_Hb delta-to-baseline values during the encoding phase showed a main effect of condition, with the whole PFC significantly less activated during encoding with music than with silence, *F*(1, 18) = 9.78, *P* = .006. For the retrieval phase, statistical analysis showed similar results for the “search for” and “free recall” tasks. Repeated-measures ANOVA on the “search for” phase revealed a main effect of condition, with higher O_2_Hb concentrations for retrieval with silence than with music (*F*(1,18) = 9.62, *P* = .006), which was also confirmed in the “free recall” phase (*F*(1,18) = 8.75, *P* = .008). The decreased PFC activation under the music retrieval condition was also supported by higher HHb values (based on the balloon model, see, e.g., [32]) for the music condition (*F*(1,18) = 6.93, *P* = .017 for the “search for” phase, *F*(1,18) = 3.56, *P* = .075 for the “free recall” phase). These results were also confirmed by paired *t*-test comparing the mean values of left and right channel for the two conditions (Figure 4).

**Figure 5:**
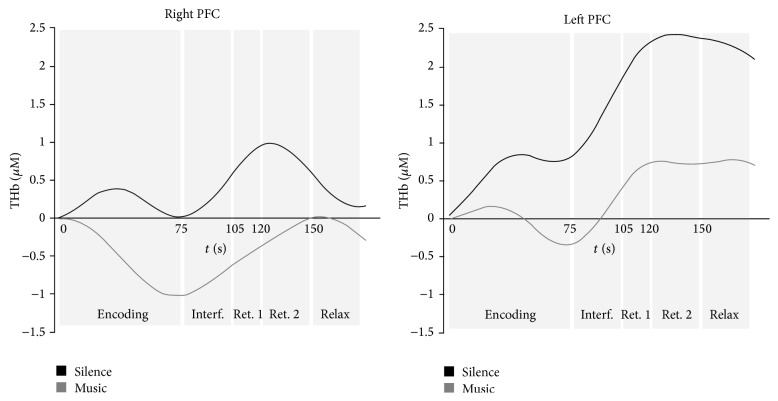
Representative single-subject time-series analysis showing THb values expressed in micromoles for silence (black lines) and music (grey lines) conditions, in right (left side) and left (right side) PFC during the encoding, interference, “search for” (ret. 1), free recall (ret. 2), and relax phases.

Time-series analysis on O_2_Hb values confirmed a main effect of the condition (*F*(1, 18) = 7.58, *P* = .013), with less PFC engagement for the music encoding and retrieval phases. This was supported by HHb time-series analyses (*F*(1,18) = 5.63, *P* = .008) which showed higher values for the music condition. A condition × laterality interaction was also found for O_2_Hb concentrations (*F*(1, 18) = 4.48, *P* = .048), suggesting higher left and right hemisphere engagement, respectively, for silence and music condition. A main effect of time was also found for both O_2_Hb (*F*(612, 34) = 19.04, *P* < .001) and HHb values (*F*(612, 34) = 6.001, *P* < .001), as shown in Figure 5.

## 4. Discussion

Extending previous studies of verbal memory encoding and music [10, 11], the present work investigated music-related episodic encoding and retrieval processes using multichannel fNIRS to monitor cortical oxygenation changes over the lateral PFC during both episodic encoding and retrieval of verbal information.

One of the main findings of this study is that activity decreased under the music condition as compared to the silence condition. In line with our previous experiments, fNIRS results during word encoding revealed that the PFC was significantly more active under the silence condition than under the music condition [10, 11]. In the light of fNIRS studies showing PFC cortex deactivation when subjects' memory performance was improved by given strategies or pharmacological stimulants [18, 19], we previously interpreted the decreased DLPFC activity during music encoding as a music-related facilitation process. More specifically, we postulated that background music, unlike silence, required less involvement of the DLPFC for organizational [17] and relational interitem processing [33] during verbal episodic encoding. The first new finding of the present study is that the decreased activity under the music condition extended across the entire lateral PFC. As shown by Figure 4, analysis on musical encoding and retrieval revealed lower O_2_Hb values in almost all channels. As mentioned in the introduction, the DLPFC and VLPFC are jointly recruited to guide the processing of interitem relational information in working memory, which promotes long-term memory for this relational information [20, 34]. In particular, VLPFC is involved in both relational and item-specific memory formation, and it seems to select goal-relevant features during episodic encoding, thus contributing to subjects' ability to select relevant item information to remember [17, 34, 35]. Although fNIRS limitations in channel localizations make it hard to specifically identify which lateral prefrontal regions are specifically involved during all over the memory task, these results suggest that that the facilitator effect of a musical background also relies on its capacity to disengage the most ventral part of the PFC from its goal-relevant selective functions. In other words, music may affect the encoding state, not only by disengaging the PFC during specific interitem relational strategies (related to DLPFC activity), but also and more generally by affecting episodic prefrontal functions, namely, the capacity to select the relevant information to remember and strategically organize it for successful memory formation.

Another crucial finding of the present study is that such PFC less activation continued during the retrieval phase. Figure 5 shows an example of time-course of the THb signal all over the block of encoding and retrieval: although the retrieval phase showed an increased activation in both conditions, especially in most ventral channels (Figure 4), this was always less pronounced for the music condition. In our opinion, this is important for two main reasons. First, the fact that the music-related PFC decrease was observed during both the “search for” phase (with background music) and the “free recall” phase (without background music) excludes the possibility that the observed PFC modulation was due to the presence of auditory stimulation rather than to a specific music effect. Secondly, music provides a less demanding way of modulating the recruitment of PFC areas crucial for long-term manipulation of information and active strategic retrieval [36–38], indicating a long-lasting effect. This is particularly important in view of the divergent results in the literature. Indeed, if music constitutes a dual-task interference [14, 15], we should have observed highest increase in neural activity for the music condition in at least one of the memory phases, as previously observed in fNIRS studies investigating dual-task situations [39, 40]. On the contrary, our results suggest that music-related memory processes rely on specific neural mechanisms underlying a less demanding prefrontal engagement throughout the stages of memory formation and retrieval.

In the light of previous fNIRS studies on memory [18, 41, 42], our results should also be viewed in terms of the contribution of fNIRS to understanding the role of PFC in long-term memory processes. Unlike our previous studies, we did not find a main effect of lateralization during word encoding. However, a more thorough time-series analysis revealed a condition × laterality interaction, suggesting higher left and right hemisphere engagement, respectively, for silence and music condition. Furthermore, a specific lateralization became evident at the retrieval “search for” phase, where we found a left and right lateralization for the silence and music condition, respectively. This condition by laterality interaction related to the presence of music when subjects tried to retrieve previously encoded words can be interpreted in the light of studies showing that the lateralization of PFC activity during retrieval depends on the availability of verbal codes, with left hemispheric involvement for verbally coded information and right hemispheric activation for nonverbally coded information [43].

Major criticism of PFC fNIRS data concerns the task-evoked changes occurring in forehead skin perfusion [44–47]; PFC activity interpretations must therefore be taken with caution. Nevertheless, our findings not only confirm that fNIRS is a good tool for noninvasive investigation of long-term memory [41, 48, 49], but can also help shed new light on music-related prefrontal episodic memory processes. In particular, we suggest that music is able to modulate all stages of memory processing in a state-dependent manner, enabling the creation of relational links that may constitute efficient mnemonic strategies, as well as the successful retrieval of relevant information. Accordingly, less PFC activity is required to put these strategies to use during either encoding or retrieval. Importantly, this explanation is supported by our behavioral results. Indeed, cluster analysis revealed that participants created significantly more chunks (i.e., formed by >2 words) during the free recall of words previously encoded with music [50]. This would indicate that subjects found it easier to adopt relational-associative strategies to create interitem (and possibly item-source) links during memory formation, which were then used as mnemonic strategies for successful retrieval. However, although we previously found that a musical background can boost item [10] and source [11] memory in recognition tasks, this was not the case for the free recall task, where no difference between music and silence was found in the number of correctly retrieved words. This suggests that behavioral paradigms often fail to characterize a reliable effect of music on memory performance, even when imaging methods are able to detect a music-related effect.

Considering many authors claiming that music hampers encoding and leads to negative results, as well as the different positive behavioral outcomes, it remains important to discuss when and how music can help memory performance. In our opinion, it is crucial to note that many kinds of paradigms using many kinds of music stimuli exist in literature and hence can lead to contrasting results. In the present study, we used a pleasant musical background with a positive emotional valence and medium arousal quality with the specific idea that music can constitute a helpful encoding context. The results can therefore be discussed in the frame of an enriched context (see, e.g., [51, 52]) given by the presence of the music, in which many mechanisms (arousal-mood modulation, emotions, and reward) intervene to orchestrate the final music-related positive effect. In this perspective, the music-dependent prefrontal modulation observed opens new questions about the interpretation of such specific PFC decreased oxygenation pattern and the related facilitation. The mechanisms underlying PFC deactivation are matter of debate and can reflect several neural processes. Some explanations can be found in regard to BOLD signal decrease, which usually corresponds to an O_2_Hb decrease and HHB increase in fNIRS signal. A BOLD decrease is usually interpreted as a deactivation that reflects a focal suppression of neural activity [53, 54] and several explanations have been proposed to clarify such deactivation. For instance, Harel et al. [55] claimed that BOLD decrease can be due to stealing of blood from less active regions into the most cerebral blood flow demanding areas. Therefore, the observed fNIRS prefrontal pattern could reflect a higher activation in other brain regions. The present multichannel fNIRS paradigm in part elucidated this question by investigating not only the DLPFC [10, 11], but also the entire PFC activity. Considering the different tasks attributed to the different regions of PFC for the episodic encoding and retrieval [17], it was reasonable to think that music could be more demanding for regions surrounding the DLPFC. Results revealed a prefrontal decrease in almost all the fNIRS channels, suggesting a huge and coherent prefrontal disengagement. However, such disengagement could be related to a greater activation in other (i.e., nonprefrontal) regions [55] that need therefore to be further investigated. Raichle and colleagues [54] proposed that such reduction of neuronal activities might be mediated through a reduction in thalamic inputs to the cortex during attention-demanding cognitive tasks or through the action of diffuse projecting systems like dopamine (see also [56]). fNIRS studies showing deactivation in nonverbal tasks (e.g., video games) have tried to interpret it in terms of attention-demanding tasks [57]. Nevertheless, this hypothesis seems in conflict with other fNIRS studies investigating prefrontal responses to attention tasks. Indeed, several authors have shown how alertness or attention states significantly increase rather than decrease PFC activation [58, 59]. Also the dopamine system can be responsible for PFC deactivation [54]. Dopamine is a neurotransmitter strongly associated with the reward system: it is released in regions such as the ventro-tegmental area (VTA), nucleus accumbens or PFC as a result of rewarding experiences such as sex, food, but also music [60, 61]. Therefore, if prefrontal less activation can be related to the action of diffuse dopamine systems and the positive effect of music may be related to reward-emotional responses as well, it is possible that music-related reward mechanisms play a crucial role in helping subjects in engaging successful verbal memory processes reflected in PFC disengagement.

Another crucial point to consider concerns the strong relationship between music and language, which has been clearly shown on both behavioral and neurophysiological level (see, e.g., [62]). It is therefore possible that, among possible general mechanisms discussed above, more language-specific processes may directly intervene during the encoding of verbal material with music. More specifically, our findings suggest that semantic-associative mechanisms may be activated more easily in presence of a musical background, thus resulting in greater clustering during the free recall task. A good example is represented by what participants reported in informal posttask metacognitive follow-up: indeed, when asked how difficult they found the task, many of the subjects suggested that music helped them in creating stories (i.e., bindings) among items and between items and music. For example, if the words “pool” and “glass” were subsequently presented and music was present, participants reported these words were easier to remember because of the creation of a little story in their mind (e.g. “*I imagined myself drinking a glass of wine while playing the pool in a bar with a jazzy atmosphere*”). In this case, the musical context may help in creating new connections between the items and the source itself, namely, new episodes that participants can then retrieve during their subjective mental “time travel” [1], as reflected by behavioral findings. Further neurophysiological investigations (e.g., investigating gamma and theta oscillations, shown to bind and temporally order perceptual and contextual representations in cortex and hippocampus [63]) could in this case elucidate possible item-source bindings processes and further research is therefore needed in this domain.

Taken together, our results overall can be seen in the general framework of the music and memory literature, supporting the idea that music can help verbal memory processes and that associative strategies facilitated by the presence of a musical background may explain memory enhancement. Given the increasing need to understand when and through which mechanisms music is able to stimulatecognitive functions, these results offer in our opinion an important contribution to the existent literature and open interesting perspectives on music-based rehabilitation programs for memory deficits.

## 5. Conclusions

The aim of this study was to focus on the prefrontal processes involved in music-related episodic memory. More specifically, we wanted to extend previous findings of prefrontal deactivation in the encoding phase of verbal material to the whole prefrontal cortex and also to the retrieval phase.

Overall, fNIRS findings show that music may specifically act and modify normal cortical activity; namely, it can entirely modulate the lateral PFC activity during both encoding and retrieval in a less demanding way. In particular, our results suggest that music-related strategic memory processes rely on specific neural mechanisms recruited throughout the stages of memory formation and retrieval. These findings are supported by behavioral evidence indicating music-related associative strategies in the recall of verbal information and offer interesting perspectives for music-based rehabilitation programs for memory deficits.
